# Respiratory physiotherapy as a tool to allow and optimize lung donation

**DOI:** 10.1016/j.rmcr.2023.101917

**Published:** 2023-10-08

**Authors:** Ricardo Miguel Rodrigues-Gomes, Lucas Lage Cendón, Rosa Martínez Rolán, Miguel Gelabert-González

**Affiliations:** a Facultade Medicina, Universidade de Santiago de Compostela, Santiago Compostela, Spain; b Hospital Álvaro Cunqueiro, Vigo, Spain; c Centro Hospitalario Santiago de Compostela, Santiago de Compostela, Spain

**Keywords:** Respiratory physiotherapy, Chest physiotherapy, Lung donation, Intensive care

## Abstract

Lung donation is complex and sometimes the secretion retention makes it impossible. We report a case of a patient that was screened as potential lung donor with PaO2/FiO2 ratio below range. As in viable patients, the respiratory physiotherapist applied a ventilator-based group of techniques with removal of huge amount of secretions and consequent increase in the PaO2/FiO2 ratio that allowed the inclusion of the patient as potentially lung donor. The protocol was applied until the extraction day, with successful extraction and transplantation. We believe that respiratory physiotherapy could have a potential role in increasing lung viability.

## 1 Introduction

Lung donation is a difficult process as patients are usually mechanically ventilated for a variable period depending on the clinical situation that is managed.

In viable ventilated patients, respiratory physiotherapy is usually applied to avoid mucus retention and its complications, being terminated when there is evidence of subject's non-viability [[1], [2], [3], [4]].

The bibliography review about respiratory physiotherapy and organ donation just produced a couple of matches: Raios et al. (2017) documented the use and percentage of chest physiotherapy techniques in potential lung donors at an Australian tertiary hospital. Another study by Lerg et al. (2017) focused on the safety of intrapulmonary percussive ventilation in potential lung donors [[4], [5], [6]].

In this case report we show the possible implications of a respiratory physiotherapy group of techniques in the management of a possible lung donor and its importance in the final outcome.

## 2 Case presentation

We report a case of a patient with severe irreversible brain damage that was evaluated as possible lung donor in asystole.

### 2.1 Patient presentation and evolution

A 43-year-old man was admitted to the ICU from the Emergency Room after resuscitated cardiopulmonary arrest.

Background: No known drug allergies; Hypertension; Diabetis Mellitus Insulin Dependent; Dislipemy; Active smoker; Myopericarditis in 2015; Chronic ischemic heart disease with ejection fraction of 45–50 %.

Current illness: The patient was brought to the Emergency Room after resuscitated in an autolytic context. Cardiopulmonary Resuscitation (CPR) maneuvers were performed with 4 adrenalines administered with need of intubation. Cardiopulmonary resuscitation lasted 20 minutes with initial rhythm in asystole which went into pulseless ventricular tachycardia after 17 minutes of advanced CPR with effective defibrillation. Upon arrival at the Emergency Room the patient presented a Glasgow Coma Scale (GCS) of 3 points with bilateral mydriasis. He was submitted to brain and cervical Computerized Tomography (CT) scan and CT angiography of the supra-aortic trunks and cerebral arteries without significant findings. The patient was finally admitted in the ICU.

Physical examination: Weight: 79kg; GCS: 3; Normohydrated; Poor peripheral perfusion; Blood pressure 100/60; Heart Rate 90 bpm; Afebrile; Petechiae on the face; Cardiac Auscultation: rhythmic, no murmurs; Pulmonary Auscultation: Anodyne; Inferior Limbs: no oedema; Symmetrical pulses. Complementary tests: Chest X-ray: no evidence of acute pleuropulmonary pathology; ECG: SR at 100 bpm, narrow QRS complex, inferior necrosis and negative inferolateral T wave; Blood analysis: normal. Arterial Blood Gas: pH 7.00, pCO2 55 mmHg, pO2 229 mmHg, HCO3 13.6 mmol/L, Lactate 11.8 mmol/L, Ionic Calcium 4.90 mg/dL, sO2 99.2 %.

Evolution: The patient was admitted to the ICU with a GCS of 3 and bilateral non-reactive mydriasis but with respiratory decompensation requiring the initiation of sedoanalgesia with fentanyl and propofol. Temperature control was performed for 48 hours. Subsequently, after the withdrawal of sedoanalgesia, the patient continued to have a poor neurological situation, so an EEG study was made, which ruled out non-convulsive status epilepticus, as well as reactivity absence. A cranial CT scan was also performed, which showed loss of supratentorial cortico-subcortical differentiation in relation to diffuse oedema. In the following days, the patient did not progress to brain death, so a new sedation window was performed, persisting in a poor neurological situation with frequent myoclonus. Life support limitation was decided, and the family accepted controlled donation. Measures were continued in the context of maintaining the donor. Ceftriaxone from the zero-pneumonia protocol was prolonged. The patient was organ donor in controlled asystole (Maastricht III) in the operating room, with Extra Corporeal Membrane Oxigenation (ECMO).

## 3 Respiratory physiotherapy intervention and evolution

To study lung quality for donation a PaO2/FiO2 ratio was assessed as defined by the European Transplantation Committee with FiO2 100 % Peep 5 and volume to normal ventilation after 10 minutes [7].

In this case, the PaO2/FiO2 ratio revealed a 245 value, being below the defined 300 cutoff for lung donation.

As in viable patients, it was requested by the medical team to be applied respiratory physiotherapy, with the protocol developed by the intensive care physiotherapist, as described.

The protocol was made based on a recent publication of Volpe et al. (2020) [2].

The ventilator hyperinflation performed for 5 minutes with volume control mandatory ventilation, respiratory rate of 15, the larger possible inspiratory time without producing auto-peep and tidal volume until 30cmH2O of plateau pressure, with the target of an expiratory bias as higher as possible (>33L/m).

The hard/brief expiratory rib cage compression (ERCC) was applied in transition from inspiration to expiration phase every 3 cycles for 5 minutes.

The Peep-Zeep with peep raised until 15cmH2O and one cycle at 0 peep, this procedure repeated 10 cycles.

During the execution of these 3 techniques, when there was evidence of secretions in larger airways, suction was applied.

The mechanical in-exsuflator (MIE) was performed in manual mode, 6 cycles per set, until secretion removal with inspiratory pressure: 40 CmH20 and expiratory pressure: −50 cmH20 [2,[8], [9], [10]].

The protocol was implemented using a step-by-step approach, with the first three techniques always being performed, and the MIE being applied only when evidence of secretions was detected after the first three steps. The evidence of secretions was assessed by the saw pattern in the ventilator curves and/or lung auscultation [2].

In this case, a Thursday, during the respiratory physiotherapy procedures, it was applied suction for 6 times with need to use the MIE with removal of abundant secretions.

**Fig. 1:**
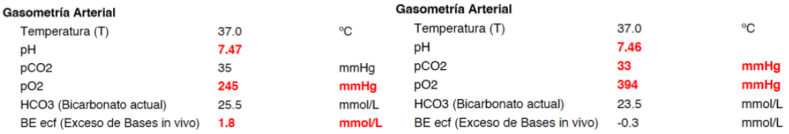
Pre and post respiratory physiotherapy blood gas.

After 30 minutes the PaO2/FiO2 ratio was retested with a 394 value, allowing a perspective of lung viability Fig. 1.

On Friday we only applied the first 3 steps, with suction performed 3 times during the respiratory physiotherapy.

There are no Physiotherapy services in our hospital during the weekends.

**Fig. 2:**
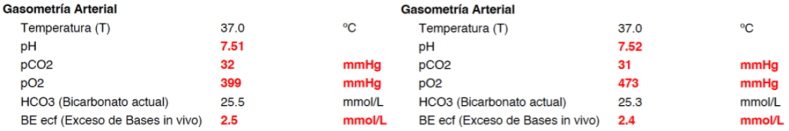
Pre and post respiratory physiotherapy in donation day.

The respiratory physiotherapy protocol was applied once per day to avoid mucus retention and 5 days after the 1st intervention (Monday), in the extraction day, before the respiratory physiotherapy the PaO2/FiO2 ratio was 399 increasing until 473 after the intervention, this day with need of suction 5 times and the use of the MIE Fig. 2.

The lungs were successfully extracted and were used for transplantation.

## 4 Discussion

**Clinical discussion:** The blood gases shown, when testing the lung quality, that the patient was not possible lung donor as the PaO2/FiO2 ratio was below range. With the multimodal respiratory physiotherapy intervention there was an improvement that allowed to consider the lung donation. By itself, with the limited number of available lung donors with quality, this fact should indicate that all potential lung donors should receive this cheap and promising intervention.

**Pathologic discussion:** As referred in the case presentation, mucus plugs are a common complication in ventilated patients. Respiratory physiotherapy is a multimodal intervention that has proved to be effective in its management in viable patients that should be continued in donors [3].

**Brief review of literature:** There is a huge lack of bibliography related with this issue as we found only the two referred studies. Our purpose with this case report is to show the potential benefit and clinical relevance that could stimulate related RCT.

**Conclusion:** Respiratory physiotherapy was effective in the optimization of this lung donor patient and may be a tool to preserve and improve lungs during the donation process.

**Figure legends:** we included figures of the blood gases pre and post respiratory physiotherapy of two different days.

## Declaration of competing interest

No conflict
